# Self-grafting-induced epigenetic changes leading to drought stress tolerance in tomato plants

**DOI:** 10.1093/dnares/dsad016

**Published:** 2023-07-15

**Authors:** Maria Isabel Fuentes-Merlos, Masaru Bamba, Shusei Sato, Atsushi Higashitani

**Affiliations:** Molecular Genetics and Physiology Laboratory, Molecular and Chemical Life Sciences, Graduate School of Life Sciences, Tohoku University, Sendai 980-8577, Japan; Instituto para la Investigación Científica y la Educación Acerca de las Enfermedades Genéticas y Metabólicas Humanas, INVEGEM, Sacatepéquez 03009, Guatemala; Symbiosis Genomics Laboratory, Ecological Development Adaptability Life Sciences, Graduate School of Life Sciences, Tohoku University, Sendai 980-8577, Japan; Symbiosis Genomics Laboratory, Ecological Development Adaptability Life Sciences, Graduate School of Life Sciences, Tohoku University, Sendai 980-8577, Japan; Molecular Genetics and Physiology Laboratory, Molecular and Chemical Life Sciences, Graduate School of Life Sciences, Tohoku University, Sendai 980-8577, Japan

**Keywords:** tomato, self-grafting, drought stress, histone modification, DNA methylation

## Abstract

Grafting is widely used as a method to increase stress tolerance in good fruiting lines of *Solanaceae* plants. However, little is known about how grafting, affects epigenetic modifications and leads to stress tolerance, especially within the same line. Here, we studied the effects of self-grafting in tomato plants on histone and DNA modifications and changes in gene expression related to drought stress. We found that at the three-leaf stage, 1 week after self-grafting, histone H3 K4 trimethylation and K27 trimethylation changes were observed in more than 500 genes each, and DNA methylation changes in more than 5,000 gene regions at the shoot apex compared to the non-grafted control. In addition, two weeks after the epigenomic changes, global expression changes continued to be observed at the shoot apex in several genes related to the metabolic process of nitrogen compounds, responses to stimulus, chromosome organization, cell cycle-related genes, and regulation of hormone levels. Finally, these grafted seedlings acquired remarkable drought tolerance, suggesting that epigenomic modifications during the wound-healing process mitigate stress tolerance in tomato plants.

## 1. Introduction

Plant grafting is a technique of primarily connecting the scion and the root ‘rootstock’.^1^ This technique is commonly used to increase stress tolerance, such as soil-borne disease and abiotic stress like drought, and increase yield by developing a vigorous rootstock in *Solanaceae* and *Cucurbitaceae* crops.^2^ To increase production and reduce losses due to environmental stress, graft vegetables with vigorous rootstock, especially from different species or hetero-grafting, to reduce the stress in the shoot.^3,4^ Tomato (*Solanum lycopersicum*) is commonly used as a model for vegetable plants because its genomic information is readily available, is easy to cultivate and graft, and is high-yielding.^5,6^ We previously found that Momotaro (Momo) homo-grafting, using the same species as a vigorous rootstock, and self-grafted, increased drought tolerance after 12 days of drought stress treatment.^7^ While it was expected in homo-grafting, we observed that self-grafting also increased drought tolerance in Momo shoots. Several differentially expressed genes were found in the shoot apical meristem; however, the mechanism behind its activation is still unclear.

Some recent studies have focused on long-distance communication of grafted junctions during the wound-graft-healing process, such as the movement of several hormones, metabolites, macromolecules, proteins, and RNA signals.^1,8,9^ The latter is highly important because mobile small RNAs associated with gene silencing are exchanged in the graft junctions and cause genetic modifications to the DNA.^9^ Hence, further analysis is required to determine whether these epigenetic changes are involved in essential traits such as stress tolerance in grafted plants. Plants must adapt to environmental stress through mechanisms that include stress acclimation by priming, usually referred to as stress memory. This phenomenon involves priming a seed or a plant to mild stress, causing the plants to store the information.^10^ However, this information enhances adaptation to subsequent stresses and accelerates the stress response. Additionally, several studies have revealed epigenetic processes in plant adaptation to abiotic and biotic stresses,^11^ including memory mechanisms involved in epigenetic changes, such as histone modification, DNA methylation, chromatin dynamics, and microRNA.^12^

This study assessed whether self-grafted tomato plants could improve drought tolerance and alter transcriptional and epigenetic changes, such as histone H3K4me3, H3K27me3, and DNA cytosine methylation, compared to non-grafted Momo. This tomato variety is mainly cultivated in Japan and is known for its sweetness, hardiness, and flavour.^13^ Our data further support the hypothesis that grafting alters epigenetic marks in the apical tissues of the scion, including the meristems, resulting in increased drought stress tolerance through stress memory within specific genes.

## 2. Material and methods

### 2.1. Plant materials, growth conditions, and grafting

Momotaro (Momo) tomato seeds were grown in a large growth cabinet (Espec Ltd., Osaka, Japan) at 28/22°C (day/night), with a 12-h photoperiod (300 μmol/m^2^/s) and 85/75% relative humidity. Seeds were obtained from Takii Seed Co., Ltd., Kyoto, Japan. The soil was kept moist by daily irrigation to avoid water logging. When Momo plants had three true leaves (three-leaf stage; 10-d old seedlings), scion and rootstock stems were cut diagonally and attached to the same or different individuals using a joint graft holder (Seem, Kita-Kyushu, Japan) for self-grafting. The plants were maintained under high humidity (85–90%) and low light for 3 days until grafting was completed. The grafted plants were then acclimated to the large growth cabinet, and healthy grafted plants at 7 days post-grafting (still at the three-leaf stage) were used for epigenetic modification analysis. In addition, Momo 10-d-old plants (three-leaf stage) were used as non-grafted controls. Shoot apexes (approximately 10 mm long) containing apical meristematic tissue were cut from each plant with a surgical scalpel and applied for chromatin immunoprecipitation sequencing (ChIP-seq) and whole-genome bisulfite sequencing (BS-seq) below.

### 2.2. ChIP-seq analyses with antibodies against histone H3 K4 trimethylation and K27 trimethylation

ChIP assay for three biological replicates per line was performed as described^14,15^ with anti-trimethyl-Histone H3-Lys4 (EDM Millipore Co., Temecula, CA, USA) and anti-trimethyl-Histone H3-Lys27 (EDM Millipore) antibodies. In addition, Dynabeads Protein A (Thermo Fisher Scientific), NucleoSpin® Gel and PCR Clean-up (Macherey-Nagel, Düren, Germany), NEBNext® Ultra™ II DNA Library Prep Kit, and NEBNext® Multiplex Oligos (New England Biolabs, Ipswich, MA, USA) were used for ChIP-DNA sequencing by MiSeq® (Illumina). Enriched peaks with significant grafting effects (*P* values of < 0.001 as calculated fold enrichment between the average of the three replicates) were analysed by the ChIP-Seq program.^16^ A significant increase in methylation by grafting was termed hypermethylation, whereas the loss was named hypomethylation.

### 2.3. Whole-genome BS-seq analyses

For cytosine-5 DNA methylation analysis, whole-genome DNA of three biological replicates per line was extracted as described,^17^ end-repaired, and ligated to the enzymatic methyl-seq (EM-seq) ligators of NEBNext® UltraTM II reagents (New England Biolabs). Libraries were prepared by NEBNext® EM-seq Kit (New England Biolabs) and sequenced using MiSeq® (Illumina). We identified the sequence variants^18^ and extracted the single nucleotide polymorphisms (SNPs).^19^ SNPs with cytosine or guanine in the control samples and thymine or adenine in the grafting samples were identified as unmethylated sites by grafting (hypomethylation), and vice versa was recognized as methylated sites (hypermethylation) with Python 3 in-house scripts and defined these as differentially methylated positions (DMPs). CG and CH (CHG, CHH) methylation contexts in which H is any base other than G, were included in the results.

### 2.4. Gene ontology categorization

The differentially methylated genes (DMGs) were analysed for gene ontology (GO) enrichment using the PANTHER 14.0 tool^20^ with a *P* value of 0.05. Only the GOs categories of interest were chosen for the family of biological processes.

### 2.5. Drought stress treatment and sample collection

In the drought stress treatment, each plant was cultured in a large growth cabinet to about 10 cm plant height and similar leaf area (Fig. 2B). The non-grafted Momo controls were at the four-leaf stage at 14-d old, the self-grafted plants were at the five-leaf stage 14 d after grafting, and the pre-drought-experienced plants (deprived water for 4 d or 7 d from the three-leaf stage and then 4 d water supply) were at the four-leaf stage. For drought treatments, each plant was not watered for 12 d under a large growth cabinet. The plants were then irrigated for 3 d, and those with new leaves emerging from the apical meristem, or from axillary buds due to loss of apical dominance, were counted as surviving plants.^7^ Meanwhile, no surviving plants showed wilted and no growth. Shoot apexes (approximately 15 mm long) containing apical meristematic tissue were collected before stress (D0) and on day 3 (D3) during drought stress treatment in three biological replications in each group.

### 2.6. RNA extraction, sequencing, data analysis, and RT-PCR

TRIzol reagent (Thermo Fisher Scientific, Waltham, MA, USA) and RNeasy Minikit (Qiagen, Hilden, Germany) were used to extract and purify total RNA. RNA pools of the three biological replicates of control and grafted (Day 0 and Day 3 of drought stress treatment) were used and sequenced by Ribo-Zero rRNA Removal Kit [Plant Leaf (Illumina, San Diego, CA, USA)], SureSelect Strand-Specific RNA Library Prep (Agilent Technologies), and NextSeq 500 (Illumina). All data analysis was done following refs ^21–24^ with ITAG4.0 tomato annotation information^5^ and featureCounts v.2.01.^25^

In addition, PrimeScript RT Reagent Kit (Takara Bio Inc., Shiga, Japan) and KAPA SYBR FAST universal (Kapa Biosystems Inc., Wilmington, MA, USA) were used to perform real-time quantitative RT-PCR. SAMs samples of control, grafted, and pre-drought stress-treated plants (on Day 0 and Day 3) were run in the CFX Connect Real-Time System (Bio-Rad Laboratories, Hercules, CA, USA) with the following cycling conditions: 3 min at 95°C, then followed by 40 cycles of 95°C for 15 s, 60°C for 30 s, and 72°C for 1 min. The RT-qPCR primers are listed in Supplementary Table S1. The internal control was set to the housekeeping gene 18S rRNA. The 2^−∆C´T^ method^26^ was used to calculate each gene’s relative expression.

## 3. Results

### 3.1. H3K4me3, H3K27me3, and DNA methylation preferentially mark genes in grafted compared with control plants

**Figure 1.**
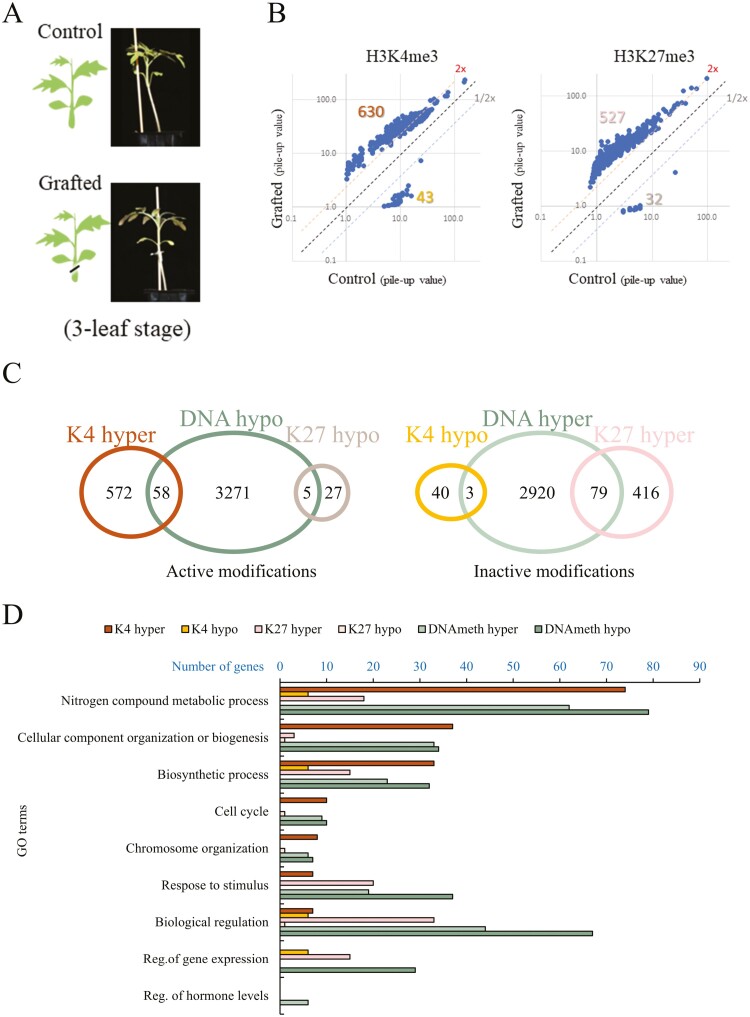
H3K4me3, H3K27me3, and DNA methylation mark different genes on grafted Momotaro (Momo) plants. (A) Phenotype of Control (ungrafted Momo) and grafted (self-grafted Momo) 7 days after grafting (three- true leaf stage). (B) Pile-up values of H3K4me3 and H3K27me3 plotted in grafted (*y* axis) and control (*x* axis) genes that showed fold enrichment as hypermethylation or hypomethylation in grafted samples. Number of differentially modified genes is indicated upper or down part of the data. Two times higher values (2×) indicate hypermethylation, while half times lower values (1/2×) indicate hypomethylation by grafting. (C) Venn diagram of the number of differentially methylated genes by grafting that had one or more than one epigenetic modification. Active modifications like H3K4me3 hypermethylation (K4 hyper), DNA hypomethylation (DNA hypo), and H3K27me3 hypomethylation (K27 hypo) where are associated with gene transcription. Inactive modifications like H3K4me3 hypomethylation (K4 hypo), DNA hypermethylation (DNA hyper), and H3K27me3 hypermethylation (K27 hyper) where are associated with gene silencing. (D) Gene ontology (GO) enrichment classification (*P* value < 0.05) of DMGs by H3K4me3 and H3K27me3 (*P* < 0.001), and DNA methylation (DMPs > |5|). The number of genes categorized under each GO term (*y* axis) is shown on the *x* axis.

To investigate whether self-grafting induces epigenetic changes in the Momo scion, ChIP- and BS-seq analyses were performed on shoot apex containing apical meristem at the three-leaf stage of ungrafted control and grafted plants. Self-grafting was done at the three-leaf stage, after which the grafted plants took a week to recover, but no new fourth leaf emerged during this wound-healing process (Fig. 1A). The grafted plants grew transiently slower than the ungrafted controls. The result of ChIP analyses showed that the selected histone H3 modifications were consistent with normal and common patterns, with H3K4me3 showing a peak at the beginning of the 5’ end of the gene and H3K27me3 indicating a uniform distribution throughout the gene body (Supplementary Fig. S1). Next, we found 630 hypermethylated and 43 hypomethylated DMGs in H3K4 and 527 hypermethylated and 32 hypomethylated DMG in H3K27 that resulted from grafting (Supplementary Fig. 1B and Tables S2 and S3). All DMGs of H3 modifications were respectively listed with fold enrichments calculated with pile-up values in each grafted vs. control (positive: fold increase, negative: fold decrease in grafted samples), in which values were normally distributed in either grafted or control samples. In addition, DNA hypermethylation and hypomethylation (at least one DMP) were observed in 3,334 and 3,002 DMGs, respectively, by grafting (Fig. 1C, Supplementary Table S4). Furthermore, Venn diagrams dividing active and inactive modifications showed that histone H3 modifications and DNA methylation did not intersect much, indicating that either epigenetic modification occurred independently in each DMG (Fig. 1C).

Gene ontology (GO) enrichment analyses of these DMGs were classified into ‘nitrogen compound metabolic process’, ‘cellular component organization or biogenesis’, ‘biosynthetic process’, ‘cell cycle’, ‘chromosome organization’, ‘response to stimulus’, ‘biological regulation’, ‘regulation of gene expression’, and ‘regulation of hormone levels’ (Fig. 1D). The enrichment was notable in higher GO categories such as biological regulation and middle categories such as response to stimulus.

### 3.2. Momo scion increases survival rate to drought stress treatment through self-grafting

**Figure 2.**
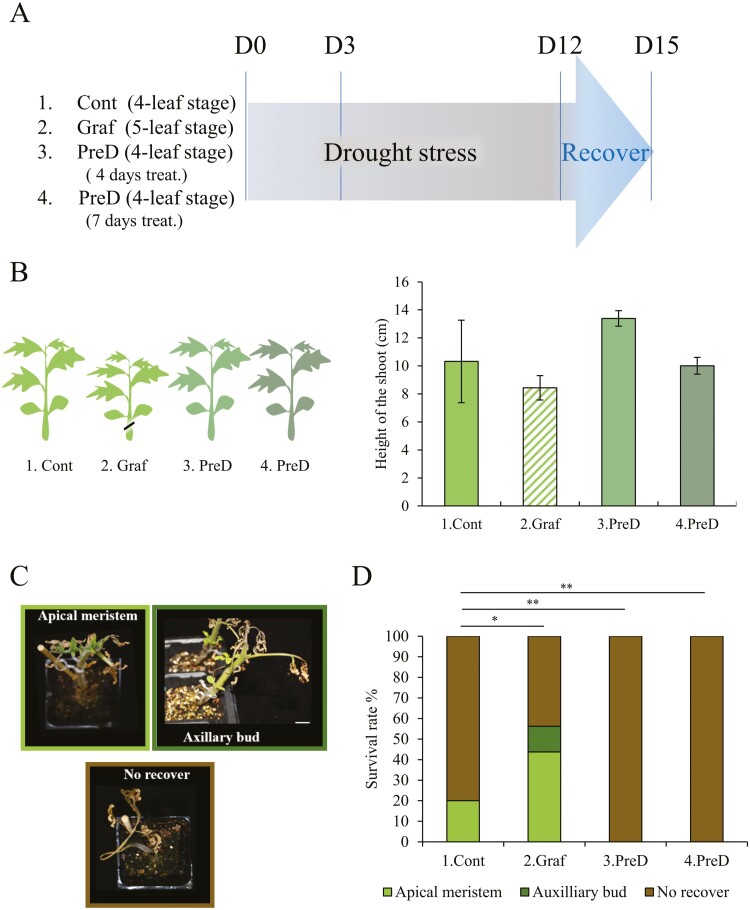
Momotaro (Momo) scion increases survival rate to 12 days drought stress treatment through grafting. (A) Sample collections for each analysis are indicated in a schematic representation for epigenetic, pre-drought, and drought stress treatment. Control and grafted indicated to Momo non-grafted and self-grafted, respectively. D0 indicates before the drought stress treatment, D3 indicates 3 days of drought stress treatment, and 7 d indicates 7 days after grafting. 1. Cont = control, 2. Graf = grafted, 3. PreD = 4d pre-drought, and 4. PreD = 7d pre-drought. (B) Graphical representation of the four Momo plant conditions used for experiment and height of the shoots in centimetres (cm) on each condition during D0. Bar represents average of three biological replicates. Error bar represents standard error. (C) Phenotype in Momo scion after 3 d of recovery of the 12 d of drought stress treatment. White bar = 1 cm. (D) Survival rate measured after 3 days of recovery from the drought stress treatment. Bar represents survival rate percentage of plants in each treatment. Asterisks denote significant differences according to chi-square test between control and treated plants, where * indicates *P* < 0.05 and ** *P* < 0.01. Control (*n* = 30), grafted (*n* = 16), 4 d pre-drought (*n* = 30), and 7 d pre-drought (*n* = 30). Black bar represents no survival individuals.

To study the effect of self-grafting and pre-drought stress-treated Momo in drought tolerance, we subjected tomato plants to 12 days of drought and 3 days of recovery (Fig. 2A). Each drought treatment was applied to plants approximately 10 cm in height and with similar leaf area, but the grafted plants had five true leaves (five-leaf stage), one leaf more than the ungrafted control due to slow stem growth but increased leaf growth (Fig. 2B). Expression levels of 13 genes involved in lowering, such as *single flower truss* (*SFT*, Solycg063100) and *falsiflora* (*FA*, Solyc03g118160),^27^ were quite low at the shoot apex of both non-grafted control and grafted plants and were not significant difference between them (Supplementary Fig. S2 and Table S5). Their low expression indicates that the floral bud transition has not yet occurred. In general, tomatoes such as Momo develop their first inflorescence on the primary stem after eight leaves have developed.^28^ Thus, most of the shoot apical tissue at the four- to five-leaf stage is in the vegetative growth stage. Control plants survival after 12 days of drought stress treatment was severely reduced to 20%, which recovered from the apical meristem area (Fig. 2C and D). The remaining 80% of the plants remained brown and never emerged with new leaves. In contrast, the self-grafted Momo was significantly more resistant to drought stress (56% recovery rate). Surviving grafted plants developed new leaves from the apical meristem (about 43%) and axillary bud (about 13%), which were thought to have reduced apical dominance. However, pre-drought treatment (4 and 7 days) did not increase tolerance but conversely decreased it (no survival individuals). These results show that drought tolerance was acquired by self-grafting without using a vigorous rootstock, while this effect was significantly different from the pre-drought stress exposure.

### 3.3. Distribution of H3K4me3, H3K27me3, and DNA methylation causes different gene expression intensity levels in grafted

**Figure 3.**
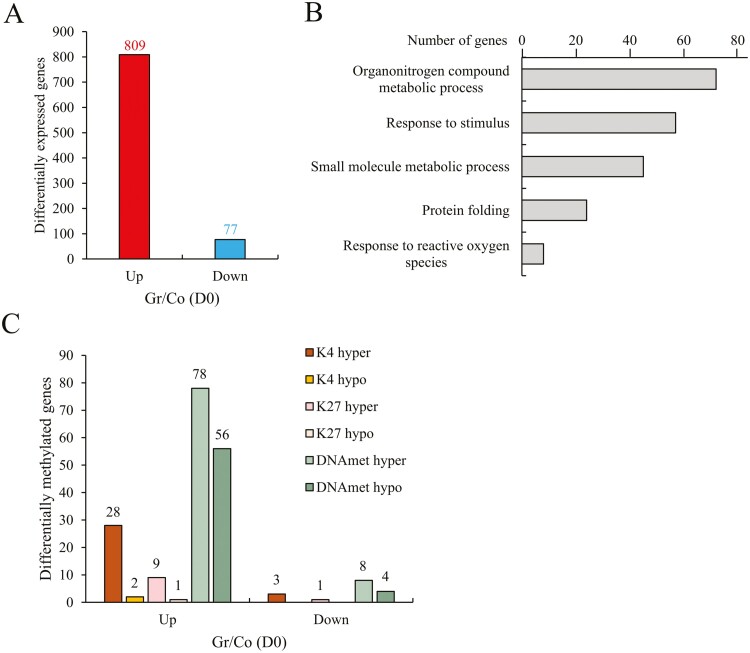
H3K4me3, H3K27me3, and DNA methylation mark different levels of gene expression between grafted and control before drought stress (D0). (A) Comparison of differentially expressed genes (DEGs) on grafted vs. control D0 (RPKM > 10). The bar represents the number of DEGs (*y* axis) in each up or down gene expression pattern (*x* axis). Gr and Co represent grafted and control, respectively. (B) Gene ontology (GO) enrichment classification of DEGs in panel (A). Selected categories with a *P* value < 0.05. (C) Differentially methylated genes (DMGs) classified on DEGs in panel (A). Each bar represents a different epigenetic modification. The *x* axis represents the up or down gene expression pattern (fold change > |2|), and *y* axis is the number of DMGs belonging to each epigenetic modification. H3K4me3, H3K27me3, and DNA methylation are abbreviated as K4, K27, and DNAmet, respectively. Hyper refers to hypermethylation, and hypo refers to hypomethylation.

In a comparison of grafted and control at D0 [Gr/Co (D0)], grafted transcriptionally upregulated 809 genes and downregulated 77 genes (Fig. 3A). Several hormone-related genes include ABA receptors, auxin, cytokinin, ethylene-responsive transcription factors, gibberellins (GA), and jasmonic acid were differentially expressed by grafting (Supplementary Table S6). In addition, DNA replication-related genes, stress-related genes, chaperone/heat shock protein genes, and reactive oxygen species-related genes were also altered (Fig. 3B). These indicate that grafting changed these gene expressions throughout the wound-healing process.

Among them, about 20% had differentially epigenetic modifications (Fig. 3C). Epigenetic modifications that generally promote transcriptional activation were found in 28 genes with H3K4me3 hypermethylation, 1 gene with H3K27me3 hypomethylation, and 56 genes with DNA hypomethylation that were upregulated by grafting (Supplementary Table S7). In contrast, epigenetic modifications that generally suppress transcriptional activity were found in two genes with H3K4me3 hypomethylation, nine genes with H3K27me3 hypermethylation, and 78 genes with DNA hypermethylation that were also upregulated by grafting (Supplementary Table S7). In the downregulated genes, three genes with H3K4me3 hypermethylation, one gene with H3K27me3 hypermethylation, eight genes with DNA hypermethylation, and four genes with DNA hypomethylation were observed by grafting (Fig. 3C, Supplementary Table S7). As typical examples shown in Table 1, the ClpB chaperone protein genes (*Solyc02g088610* and *Solyc06g011370*), auxin efflux carrier family protein (*Solyc10g074790*), S-adenosylmethionine decarboxylase (*Solyc02g150147*), cytokinin riboside 5-monophosphate phosphoribohydrolase (*Solyc04g081290*), and abscisic acid (ABA) receptor PYL10 (*Solyc01g095700*) were transcriptionally upregulated by grafting [Gr/Co (D0)] at D0 steady-state levels.

**Table 1.** Classification of differentially methylated genes by H3K4me3, H3K27me3, and cytosine-5 DNA methylation in grafted and gene expression on control (Co) and grafted (Gr) on before (D0) and during drought stress (D3) treatment. Histone modification presented as fold enrichment (*P* < 0.001) and DNA methylation as differentially methylated position (DMPs > |1|). Positive values represent hypermethylation (hyper), and negative values represent hypomethylation (hypo). Gene expressions are normalized in RPKM (Reads per Kilobase of transcript, per Million mapped reads). Differential expression indicates if fold change (FC) between grafted and control is up (upregulation), down (downregulation), or not change (nc) by grafting

Gene ID	Annotation	Epigenetic mark	Fold enrichment or DMPs	Gene expression (RPKM)				Differential expression	
				Co D0	Co D3	Gr D0	Gr D3	FC D0	FC D3
Solyc01g090340	Ethylene-responsive transcription factor 2	H3K4me3 hyper	2.1	20.5	18.8	46.4	39.0	Up	Up
Solyc01g104650	ABSCISIC ACID-INSENSITIVE 5-like protein 5		2.6	1.9	21.2	3.8	13.8	Up	nc
Solyc02g088610	LeHSP110/ClpB heat shock protein		3.4	13.4	80.5	27.0	21.8	Up	Down
Solyc03g005780	Chlorophyll a-b binding protein, chloroplastic		2.3	668.0	1.4	335.5	34.5	nc	Up
Solyc06g076640	Tubulin beta chain		2.3	151.4	14.9	189.0	31.1	nc	Up
Solyc07g043560	Heat shock protein 70 kDa		1.7	15.6	9.2	42.7	9.1	Up	nc
Solyc07g041720	Germin-like protein	H3K27me3 hyper	4.3	570.1	0.7	264.3	66.6	Down	Up
Solyc02g150147	S-adenosylmethionine decarboxylase	DNAmeth hyper	5	10.1	258.5	83.1	91.3	Up	Down
Solyc03g111260	Ribonuclease J		5	28.1	7.8	11.4	27.5	Down	Up
Solyc04g081290	Cytokinin riboside 5-monophosphate phosphoribohydrolase		6	4.4	197.9	13.7	30.1	Up	Down
Solyc06g011370	Chaperone protein ClpB		7	6.1	44.0	15.6	17.9	Up	Down
Solyc07g054460	H/ACA ribonucleoprotein complex non-core subunit NAF1		11	12.1	4.7	5.3	7.7	Down	nc
Solyc10g074790	Auxin efflux carrier family protein	DNAmeth hypo	-4	5.5	35.8	11.8	24.0	Up	nc
Solyc09g008670	threonine deaminase		-6	7.8	43.4	123.6	108.6	Up	Up
Solyc10g086150	RNA-binding protein		-16	350.7	38.9	131.6	123.2	Down	Up
Solyc11g071640	Glycosyl hydrolase family protein		-6	134.3	2.1	84.7	10.5	Down	Up
Solyc01g095700	Abscisic acid receptor PYL10	DNA meth hyper / hypo	1 / -2	6.4	15.6	15.7	22.1	Up	nc
Solyc07g048110	ERD (Early-responsive to dehydration stress) family protein		1 / -4	3.6	0.8	4.2	2.2	nc	Up

### 3.4. Graft-induced changes in gene expression with epigenetic modifications do not change similarly in response to pre-drought stress

**Figure 4.**
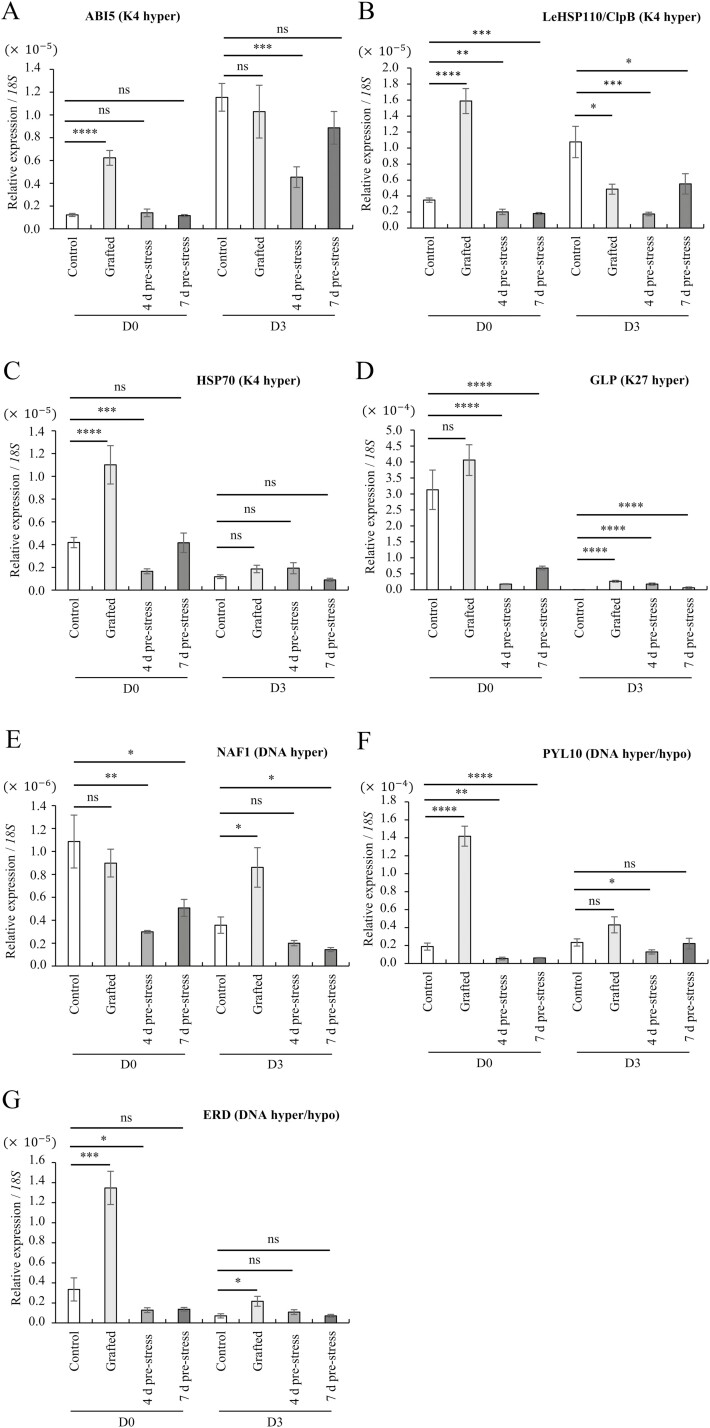
Expression level of four selected genes with epigenetic modification in grafted samples. Genes with H3K4me3 hypermethylation: (A) abscisic acid-insensitive 5-like protein 5 (*ABI5*), (B) LeHSP110/ClpB heat shock protein (*LeHSP110/ClpB*), and (C) heat shock protein 70 kDa (*HSP70*). Gene with H3K27me3 hypermethylation: (D) Germin-like protein (*GLP*). Gene with DNA hypermethylation: (E) H/ACA ribonucleoprotein complex non-core subunit NAF1 (*NAF1*). Gene with DNA hyper and hypomethylation: (F) abscisic acid receptor PYL10 (*PYL10*) and (G) early-responsive to dehydration stress family protein (*ERD*). The *y* axis shows the average expression level from control (Momo), grafted (self-grafted Momo), 4 d, and 7 d pre-stress drought treatment (non-grafted Momo). Asterisks show significant differences between the control and each treated plant according to the t-test (Mann–Whitney test), where * denotes *P* < 0.05, ** *P* < 0.01, *** *P* < 0.001, and *P* < 0.0001. The error bard displays the three biological replicates’ standard error.

To study whether gene expression differs between grafted (drought tolerance) and pre-drought stress treatment (drought susceptible), RT-PCR analyses were performed on a subset of expression-altered genes with an epigenetic modification in the grafted samples (selected from Table 1). An ABA-insensitive 5-like protein 5 or *ABI5* (*Solyc01g104650*) and two heat shock protein (HSP) genes, *LeHSP110/ClpB* (*Solyc02g088610*) and *HSP70* kDa (*Solyc07g043560*), were hypermethylated with H3K4me3 and were significantly upregulated in grafted samples under D0 conditions (Fig. 4A–C). In contrast, pre-drought stress treatment was not induced or slightly reduced compared to control. The germin-like protein (*GLP*) (*Solyc04g041720*), an H3K27me3 hypermethylated gene, showed a significant reduction in gene expression in the pre-drought-treated samples at D0 (Fig. 4D). During D3 drought stress, grafted samples showed less reduction in gene expression. GLPs are involved in various abiotic and biotic stress responses in plants.^29^ A similar pattern was observed in the H/ACA ribonucleoprotein complex non-core subunit *NAF1* (*Solyc07g054460*), which increased 11 DMPs in the grafted samples (Fig. 4E). *NAF1* is essential for several RNA processing and ribosome biogenesis.^30–32^ Several genes showed more than one epigenetic modification, such as ABA *PYL10* (*Solyc01g095700*) and *ERD* or early-responsive to dehydration stress (*Solyc07g048110*), were modified through hyper and hypomethylation of DNA (Fig. 4F and G). Both genes showed significantly increased gene expression in grafted samples under D0 conditions. These results clearly show that the expression of the molecular chaperon *HSPs*, drought-responsive genes like *ABI5, PYL10,* and *ERD,* and growth-critical genes, *GLPs* and *NAF1* is altered by grafting but not our pre-drought stress treatments. These differences would be directly related to the acquisition of drought tolerance in self-grafted tomatoes.

## 4. Discussion

A previous study found that DNA methylation changes by grafting were related to rootstock-scion interactions in hetero-grafting tomato plants.^33^ In addition, there are increasing reports of similar DNA methylation and non-coding RNA changes by grafting in some hetero-grafting plants.^34^ We here found that even self-grafted tomato plants that do not use vigorous rootstocks increase drought tolerance (Fig. 2D). In addition to changes in gene expression, epigenetic changes due to H3K4me3, H3K27me3, and DNA methylation occur in at least a total of 20% of genes of grafted plants (Fig. 3A and C). DNA methylation in plant genomes shows the highest in the CG context but also exists in non-CG methylation, where is highly stable between tissues.^35,36^ In tomato leaf, the percent of CG, CHG, and CHH is 84.1, 54.8, and 8.4, respectively.^36^ In our analysis, we englobe the methylations type in control and grafted, only comparing the DMGs in any context due to the difference of Momotaro and reference tomato background.

Selected histone modifications have been reported to play a role in drought stress memory in several genes in *Arabidopsis thaliana*.^37,38^ DNA methylation has also been reported to function in drought adaptation of the rice genome.^39,40^ We observed that the H3K4me3, H3K27me3, and DNA methylation DMGs belong to several pathways (Fig. 1D). The GO analysis of these DMGs includes ‘response to stimulus’, ‘cell cycle’, and ‘regulation of hormone levels’. Several phytohormones, such as abscisic acid, ethylene, auxin, cytokinin, and gibberellin, have been essential for stress response and graft union.^41^ Many of these hormone-related genes were found in DMGs and DEGs in the grafted plants in this study (Supplementary Tables S2–S4, S6, and S7). Abscisic acid is not required for the graft reconnection process, and its activation occurs in early response to wounding.^42–44^ During water deficit stress, ABA is important for plant adaptation through the induction of stomatal closure as a measurement of internal water control.^45^ A study found a correlation between histone modification and the ABA pathway, affecting the gene expression of dehydration-responsive genes.^46^ In our data, we observed that genes related to the ABA pathway, *ABI5* and *PYL10*, and *ERD* were activated by grafting (Fig. 4A, F, and G) under normal conditions. Those genes may be induced by H3K4me3 and DNA hypomethylation. *ABI5* is a bZIP transcription factor that functions in the centre of the ABA signalling where the overexpression of Arabidopsis orthologue ABI5 increases sensitivity to ABA and sugar levels.^47,48^ The overexpression of ABA *PYL9* in Arabidopsis (orthologue with *PYL10*) has been demonstrated to confer drought resistance; however, it also induces leaf senescence.^49^

Auxin and cytokinin are essential for graft reconnection.^9,50^ In addition, gibberellin is activated during grafting and plays a role in expanding the cortex cells of the vascular tissue formed during the graft-healing process.^42^ Like the ERF family, several transcription factors, such as apetala 2/ethylene response factor (AP2/ERF) family, play an essential role in growth and development, stress tolerance, and hormonal signal transduction.^51,52^ These findings suggest that hormone signalling contributes to stress tolerance through the epigenetic modification caused by grafting. While several of these hormones have an essential role in growth and development, they can also aid in coordinating the plant to the stress response. It is well known that hormones and epigenetic regulation have a crosstalk mechanism where the hormone can directly affect the epigenetic modifier activity, or the epigenetic modification targets genes related to the hormone pathway.^53^

In addition, the apical meristem reprograms stem cells to differentiate into new tissues.^54,55^ Some of the expressed genes whose effect was less affected by grafting during D3 belonged to the GO term ‘cell cycle’. The inhibition of the cell cycle reduces plant growth, which is a stress response mechanism.^56,57^ Cell cycle regulation involves cell division and expansion regulated by chromatin modifiers.^58,59^ Several DMGs classified as ‘cell cycle’ were marked by H3K4me3 and DNA hypomethylation. Cell cycle-related genes are important during the wound and regeneration process in the grafting area. Several studies have demonstrated that cellular reprogramming is regulated by several histone modifications and DNA methylation, causing gene expression changes after wounding.^44,60^

Grafting is assumed to cause priming drought stress due to the lack of water supply caused by xylem disconnection. On the other hand, 4- and 7-day pre-drought stress treatments in this study were expected to have similar drought tolerance effects, but the results were the opposite and became more sensitive. It may be due to different root damage between grafting and pre-drought treatment. In Arabidopsis, drought stress has been reported to cause epigenetic changes within 5 h,^37^ and multiple air-dryings for 2 h also acquire a memory of drought stress with epigenetic modifications.^46^ In contrast, we found here that even self-grafting is a simple and reproducible procedure for acquiring stress memory with epigenetic modifications. Recently, similar gene expression changes were found in a homo-grafted tomato with vigorous rootstock.^7^ However, self-grafting through wounding could have strengthened the root system. One mechanism of wounding or mechanical stress that induces changes in plants is thigmomorphogenesis, where the signalling pathways of phytohormones are implicated and thus may trigger potential responses to stress.^61^

Combined with this study, there are at least two grafting effects, stress memory due to the wound-healing process and stress tolerance acquired by vigorous rootstock combination. We are now attempting to consider other epigenetic mechanisms like small RNAs and histone modifications such as acetylation involved in grafting and stress memory. In conclusion, self-grafting generated several changes in the epigenomics of the tomato scion, where some DMG influenced some DEGs. In addition, we observed that H3K4me3, H3K27me3, and DNA methylation DMGs in several pathways could significantly regulate drought stress tolerance.

## Supplementary Material

dsad016_suppl_Supplementary_Materials

## Data Availability

The data for this study are available from the authors upon request. All global gene expression, ChIP-seq and Bisulfite-seq data are deposited in the BioProject database as the accession number of PRJDB13627.
