# Supplementary figures and images for: Self-grafting-induced epigenetic changes leading to drought stress tolerance in tomato plants
Source: DNA Res. 2023 Jul 15;30(4):dsad016. doi: 10.1093/dnares/dsad016 (PMC10368339; doi:10.1093/dnares/dsad016)

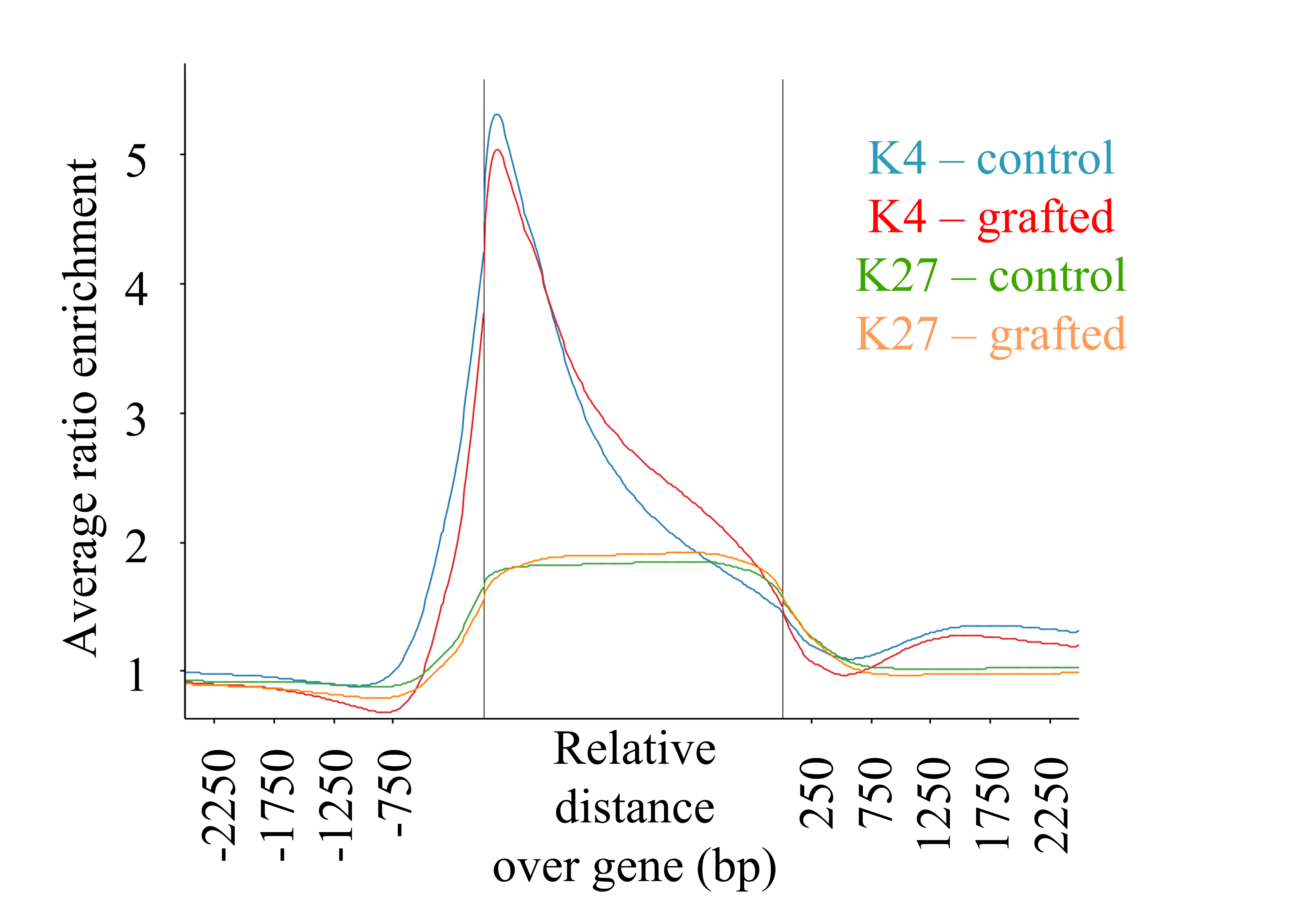

Supplement: dsad016_suppl_Supplementary_Materials [file dsad016_suppl_supplementary_materials.zip › Figure S1.tif]

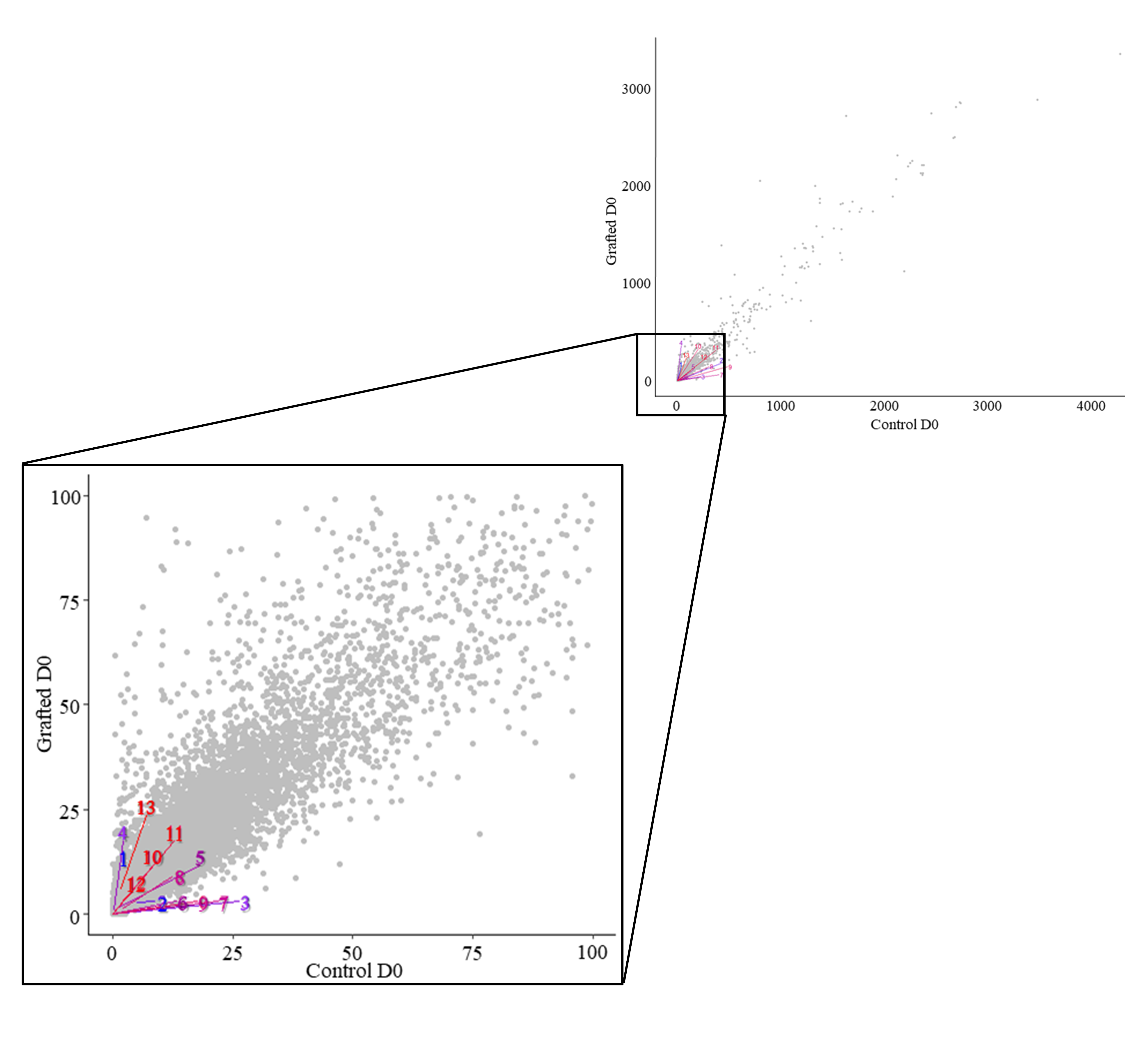

Supplement: dsad016_suppl_Supplementary_Materials [file dsad016_suppl_supplementary_materials.zip › Figure S2 first.tif]
